# Supplementary material for: Latent neural dynamics encode temporal context in speech
Source: Hear Res. Author manuscript; Available in PMC 2024 Jun 17. (PMC11182421; doi:10.1016/j.heares.2023.108838)
Supplement: Supplementary materials [file NIHMS1997385-supplement-Supplementary_materials.pdf]

Supplementary Material

Table S1. Clinical and demographic details for subjects. All subjects were left-language-dominant and were implanted over the left hemisphere.

Subject ID	Age	Sex	Handedness	Epilepsy focus
SL01	44	M	R	Left posterior STG
SL02	19	M	R	Left anterior frontal lobe
SL03		M		Left anterior temporal lobe
SL04	32	F	R	Left anterior temporal lobe
SL05	25	F	L	Left medial temporal lobe
SL06	31	F	R	Left hippocampus/anterior lateral temporal
SL07	20	F	R	Left hippocampus
SL08	60	M	R (converted from L)	Left mesial temporal structures
SL09	26	M	R	Left mesial and anterior lateral temporal cortex
SL10	22	M	R	Left anterior temporal lobe
SL11	31	F	R	Left hippocampus/amygdala

Table S2. Center frequencies and bandwidths for the filter bank used in high gamma amplitude estimation. Bandwidth is defined as the standard deviation of the Gaussian filter in the frequency domain. All values in Hz (s^{-1}).

Center Frequency	72.0	79.5	87.8	96.9	107.0	118.1	130.4	144.0
Bandwidth	4.7	4.9	5.2	5.4	5.7	6.0	6.3	6.6

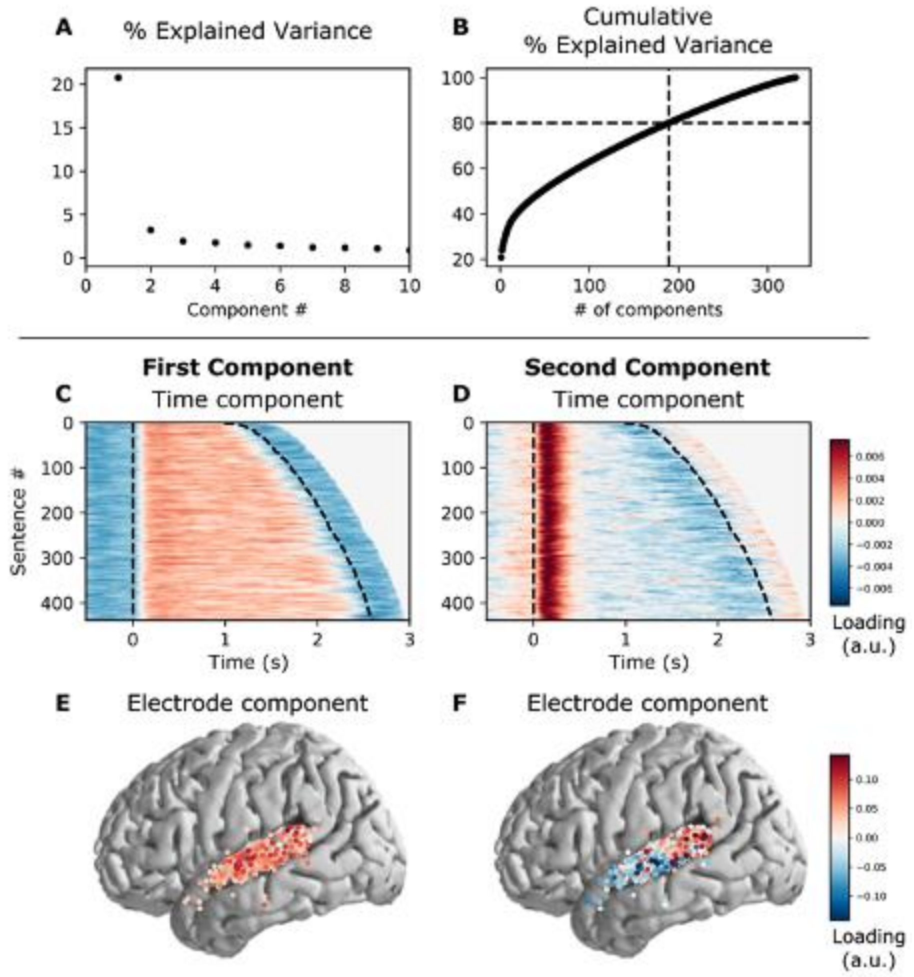

Figure S1: PCA partitions the high gamma activity across speech-responsive electrodes into a posterior onset response and a spatially widespread sustained response (see Section 3.12). A: The percent explained variance of the principal components. B: The cumulative percent explained variance. Note that 189 dimensions are required to capture 80% of the variance in the high gamma activity. C: The timecourse of the first component, aligned to sentence onset. Dashed lines indicate the start and end of the sentence stimulus, and the sentences have been ordered by their duration. This component has sustained responses, in the sense that the activity is high during the entire stimulus. D: The timecourse of the second component, aligned to sentence onset. This component has onset responses, in the sense that there is a short positive transient immediately after sentence onset. E: The spatial support of the first component. This component is spatially spread out over all of STG. F: The spatial support of the second component. This component is spatially divided, with strong positive weights over posterior STG.

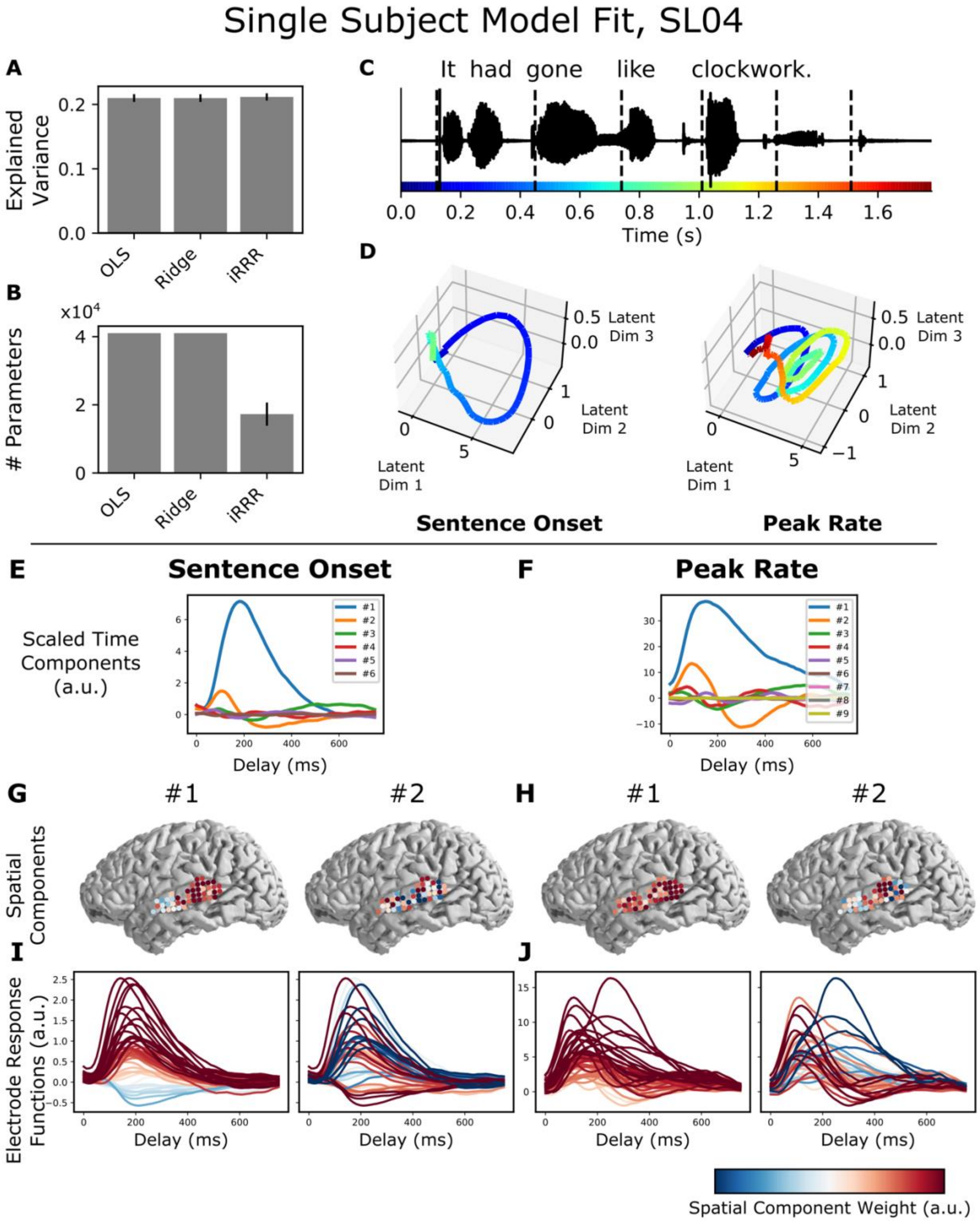

Figure S2: The model yields similar results when computed for a single subject, SL04 (see Section 3.13). A,B: iRRR outperforms OLS and ridge regression in a single subject. Panels show the total explained variance and the total number of parameters as in Figure 1 C and E, respectively. iRRR explains approximately the same variance using far fewer parameters, as in the across-subject case. C,D: Feature latent states in the single subject have rotational dynamics. Panels show the acoustic waveform and the top three dimensions of the sentence onset and peak rate latent states as in Figure 3 A, D, and E, respectively.

Rotational dynamics are evident. E,F,G,H,I,J: The single-subject model fit captures known response differences between pSTG and mSTG. Panels show the iRRR model fits for the sentence onset and peak rate features, as in Figure 2. The first dimension of the Sentence Onset response marks electrodes with the largest amplitude responses, which occur primarily over pSTG. Peak rate responses are spread over both mSTG and pSTG, as observed in the across-subject model.
